# Parallel study of transient dosing of antibiotics in a microfluidic device

**DOI:** 10.1063/5.0091704

**Published:** 2022-08-01

**Authors:** Darius G. Rackus, Petra Jusková, Fumiaki Yokoyama, Petra S. Dittrich

**Affiliations:** Department of Biosystems Science and Engineering, ETH Zurich, Mattenstrasse 26, 4058 Basel, Switzerland

## Abstract

Microfluidic tools are well suited for studying bacteria as they enable the analysis of small colonies or single cells. However, current techniques for studying bacterial response to antibiotics are largely limited to static dosing. Here, we describe a microfluidic device and a method for entrapping and cultivating bacteria in hydrogel plugs. Ring-shaped isolation valves are used to define the shape of the plugs and also to control exposure of the plugs to the surrounding medium. We demonstrate bacterial cultivation, determination of the minimum inhibitory concentration of an antibiotic, and transient dosing of an antibiotic at sub-1-h doses. The transient dosing experiments reveal that at dose durations on the order of minutes, ampicillin's bactericidal effect has both a time and concentration dependency.

## INTRODUCTION

A range of microfluidic devices and methods have been developed to cultivate and study bacteria^1,2^ as micro- (and nano-) structures are well-suited to the tasks of capturing and manipulating small, highly motile, and rapidly multiplying bacteria. Some strategies include confining bacteria to growth in either one^3,4^ or two dimensions.^5^ So-called “mother machine” devices for one-dimensional growth have been particularly useful for studying cell dynamics and tracking lineages^4^ and can even be used for single-cell phenotypic and genotypic analysis of heterogeneous populations.^6^ Bacteria can also be embedded within a hydrogel matrix,^7^ which restricts motility and helps to contain progeny while still allowing for the delivery of nutrients through diffusion. Another approach has been to encapsulate bacteria within droplets,^8^ which has the advantage of generating thousands of individual cultivation chambers with general ease. However, it can be difficult to alter the contents of the droplets, thus making it difficult to perform experiments that investigate transient dosing of compounds. One method to overcome this is the use of bacteria encapsulated in hydrogel droplets. After gelification, the hydrogel droplets (or beads) can be transferred in and out of different solutions and the beads are also compatible with flow cytometry.^9^ While flow cytometry offers incredibly high-throughput analysis, it only offers a single snapshot in time and does not easily lend itself to tracking individual cells or colonies over time. Despite the many proposed microfluidic devices, those that are simple and easy to use tend to not be suitable for multiplexing. On the other hand, those that are powerful at multiplexing across different conditions and in a time-dependent manner tend to be too complex. Therefore, a middle ground with modest multiplexing capabilities arising from minimal complexity would be useful.

Lately, much attention has been given to the development of miniaturized platforms for rapid testing of antibiotic resistances and susceptibility.^10–13^ For instance, microfluidic tools for bacterial classification^14,15^ as well as studying bacteria under continuous concentration gradients^16^ and combinations of antibiotics^17,18^ have been reported. These tools can be useful in minimizing antibiotic resistance by reducing the time that inappropriate or broad-spectrum antibiotic therapies are administered. However, these tools study bacterial death under steady-state dosing, which does not necessarily represent *in vivo* conditions where antibiotic concentrations may fluctuate.^19^ Most importantly, the persistence of bacteria against antibiotics has been linked with transient exposure to antibiotics.^20^ To account for the concentration profiles of antibiotics *in vivo*, pharmacokinetic/pharmacodynamic models have been developed.^21^ Among them, so-called hollow fiber infection models enable long-term monitoring of drug absorption and elimination *in vitro.* Cells are retained in a compartment of 10–20 ml and medium, and the drug is supplied via multiple porous fibers. The compartments are, however, large scale, i.e., 10–20 ml in volume, and pumping rates of 60–120 ml are applied.^22,23^ In addition, the hollow fiber cartridges are usually not suitable for observing cells under a microscope.

Here, we present a microfluidic device for bacteria cultivation, capable of generating short-pulse dosing profiles of antibiotics. Ring-shaped pneumatic isolation valves define individual culture chambers of approximately 400 pl in volume and control exposure of the chamber's contents to the surrounding media. Similar isolation valve devices have been previously used where hydrodynamic traps have captured mammalian cells reliably.^17–19^ However, hydrodynamic traps are less suitable for trapping the much smaller bacterial cells and challenging to fabricate with sub-micrometer gap sizes as required for bacteria.^24^

Therefore, we use here plugs of agarose hydrogel formed within the isolation valves to immobilize bacteria within the culture chamber and prevent their escape during media exchange. These plugs entrap the bacteria but enable exchange of nutrients, antibiotics, stains, and potentially other reagents. We demonstrate the ability to cultivate *Escherichia coli* in hydrogel plugs in the microfluidic device and measured the steady-state metric of minimum inhibitory concentration (MIC) for ampicillin with *E. coli* K12 MG1655 (pSEVA271-*sfgfp*). However, the true value of the presented microfluidic device is its ability to administer transient and time-dependent dosing profiles, which we demonstrate by supplying short-term pulses at concentrations above the MIC. Although ampicillin's bactericidal activity is characterized as being time-dependent in the steady state when dosed at concentrations greater than the MIC, we observed that, for short pulses, its activity appears to be dependent on duration and concentration.

## RESULTS AND DISCUSSION

**FIG. 1.**
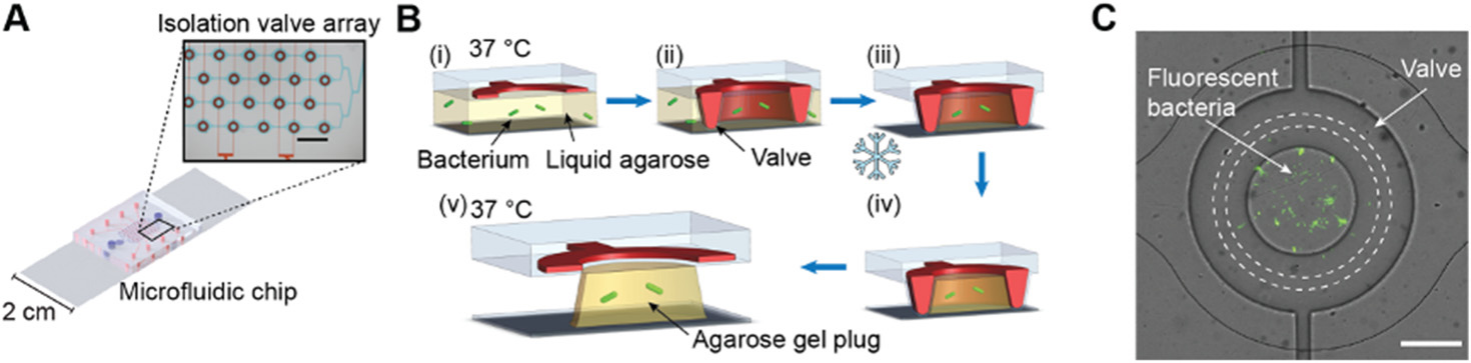
Microfluidic device for transient dosing of antibiotics. (a) A computer rendering of the microfluidic chip shows the 64 ring-shaped isolation valves (red) controlled by eight individual pressure lines and the fluidic channel (blue). The micrograph shows the isolation valves filled with red food dye and fluidic channels (height 17 *μ*m) filled with blue food dye. Scale bar: 1 mm. (b) Schematic (side view; not to scale) of *in situ* hydrogel formation in five steps: (i) a mixture of bacteria (green) and agarose gel (yellow) is loaded into the microfluidic chip; (ii) isolation valves are engaged; (iii) agarose is washed away and the chip is placed on ice for gelation; (iv) the agarose is gelled and media (not illustrated) can be replaced; and (v) isolation valves are opened to add antibiotics or medium to the hydrogel plugs. (c) Composite micrograph of an isolation valve containing *sfgfp*-expressing *E. coli* immobilized in an agarose plug. The dotted lines indicate the region where the isolation valve makes contact with the glass slide. Scale bar: 100 *μ*m.

The administration of time-dependent dosing profiles of antibiotics to populations of bacteria requires the ability to exchange media without dislodging the bacteria. Our method, outlined in Fig. 1(a), uses a microfluidic device to cultivate bacteria in addressable isolation valves and control the exposure of the bacteria to the antibiotic. The device comprises 64 individual isolation valves that each confine a volume of approximately 400 pl and that are addressable in groups of eight, through connections to eight pressure lines. Bacteria were immobilized in hydrogel plugs formed within the valves to prevent them from dislodging during experiments. We used ultra-low gelling temperature agarose because it allowed us to load the bacteria pre-mixed with the hydrogel into the microfluidic device; the risk of cell damage associated with UV-crosslinking or the added complexity of additional crosslinking reagents was avoided. Individual hydrogel plugs were formed within the isolation valves as illustrated in Fig. 1(b). A mixture of bacteria and liquid agarose is loaded into the microfluidic device. The isolation valves are then closed while excess agarose is cleared out with wash buffer before placing the microfluidic device on ice. After gelation, the valves can be opened, revealing a plug of agarose with immobilized bacteria. The valve can be closed forming a contact seal with the glass as shown in Fig. 1(c) to isolate the cells during experiments.

**FIG. 2.**
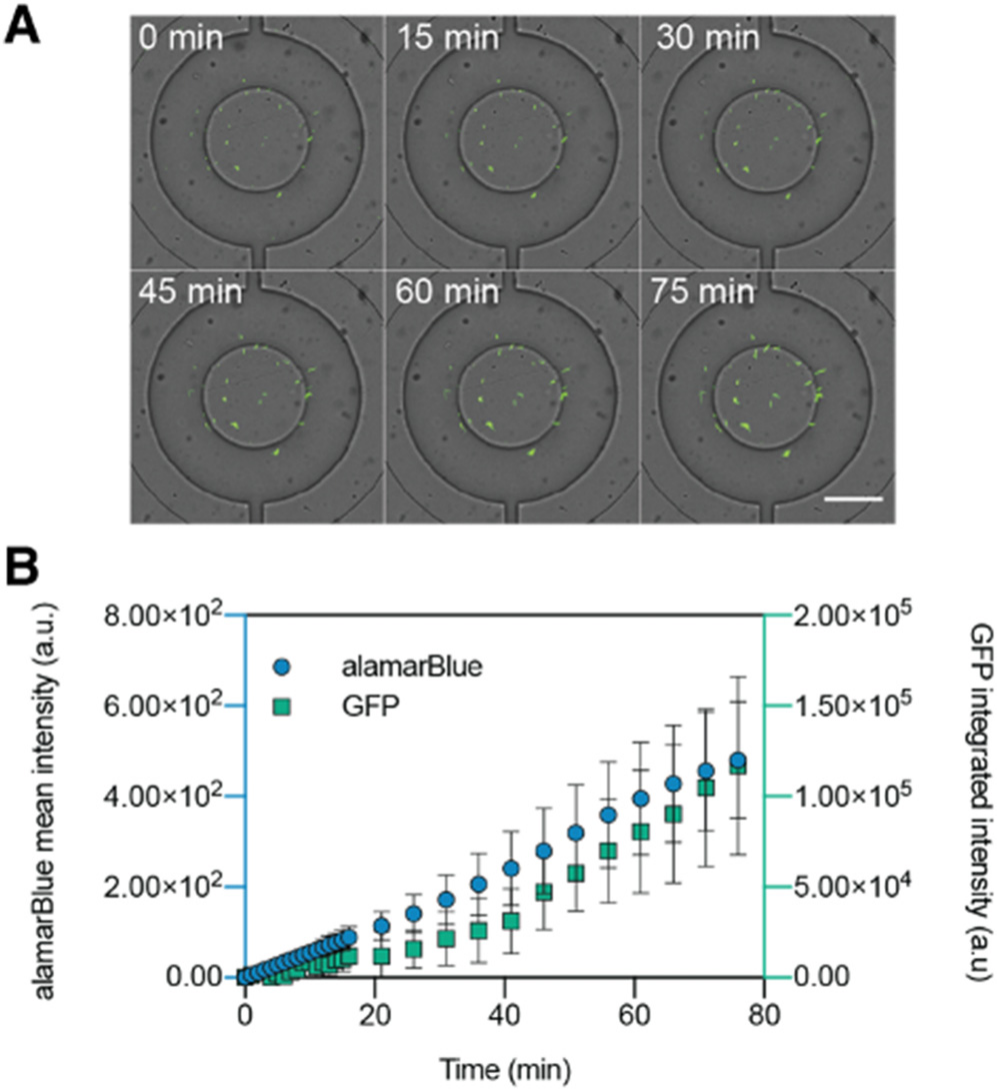
Cultivation of *E. coli* in hydrogel plugs. (a) Composite micrograph of *E. coli* cultivated in a 2.85% (w/v) agarose plug in an isolation valve. Note that the valves have a conical shape so some cells appear as if underneath the valve. Scale bar: 100 *μ*m. (b) Viability and growth of *E. coli* as monitored simultaneously by fluorescence of reduced alamarBlue reagent (resorufin) and GFP. Error bars: ±1 standard deviation, *n* = 9.

The agarose gel plugs do not interfere with bacteria cultivation within the isolation valve and they keep the bacteria in place when the valves are opened for reagent exchange with the surrounding channel. Growth of *sfgfp*-expressing *E. coli* within agarose plugs was monitored by fluorescence microscopy [Fig. 2(a)]. *E. coli* appear to remain in the position where they were initially seeded during gelation, and colonies grow from these individual immobilized bacteria. Because of the agarose gel's porosity, it is possible to introduce reagents from the surrounding microchannel by opening the isolation valves. We monitored bacterial growth using the alamarBlue viability reagent (resazurin) and sfGFP fluorescence, which served as an arbiter for biomass^13–25^ [Fig. 2(b)]. Both alamarBlue and sfGFP fluorescence track together demonstrate the viability of the growing *E. coli* population. For subsequent experiments, sfGFP fluorescence was chosen as an indicator of *E. coli* growth.

**FIG. 3.**
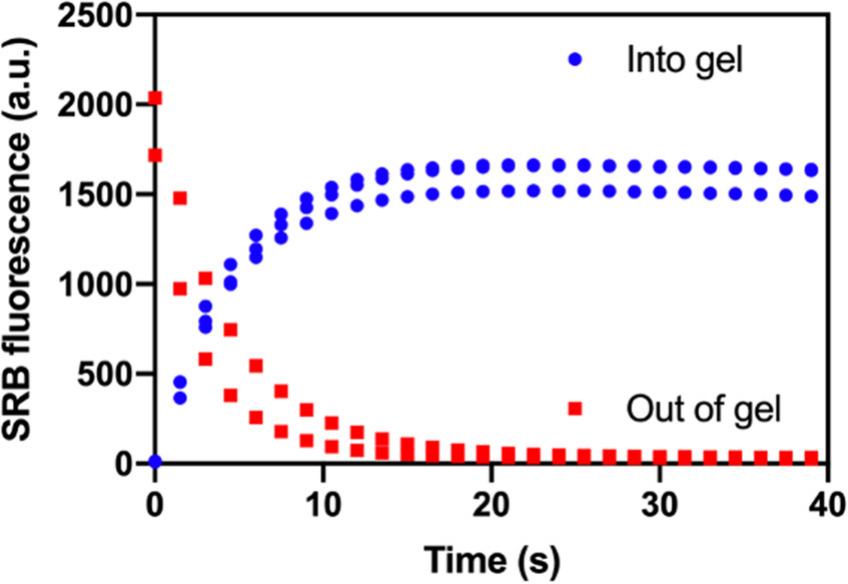
Diffusion of SRB into (blue, *n* = 3) and out of (red, *n* = 2) hydrogel plugs. Fluorescence measured as mean intensity within the isolation valve every 1.5 s for 37.5 s.

The ability to generate time-dependent dosing profiles can be useful in elucidating mechanisms of antibiotic persistence. We sought to create such profiles in our microfluidic device by using isolation valves to control the exposure of hydrogel plugs to the surrounding medium. Antibiotic exposure and clearance are dependent on diffusion in and out of the hydrogel. Therefore, we characterized diffusion in and out of the agarose plugs by monitoring the increase or decrease in fluorescence intensity of a fluorophore into or out of the plug, respectively. Sulforhodamine B (SRB), 580.65 Da, is of comparable size to the antibiotic ampicillin, 349.41 Da, and was chosen to give an indication of antibiotic transport into and out of hydrogel plugs. Figure 3 reports the fluorescence intensity of SRB within the isolation valve as it either diffuses into or out of the hydrogel. Maximum and minimum fluorescence intensities were observed after approximately 20 s. For later experiments, we used 1 min as the minimum time for keeping the isolation valve open when adding or removing antibiotics.

**FIG. 4.**
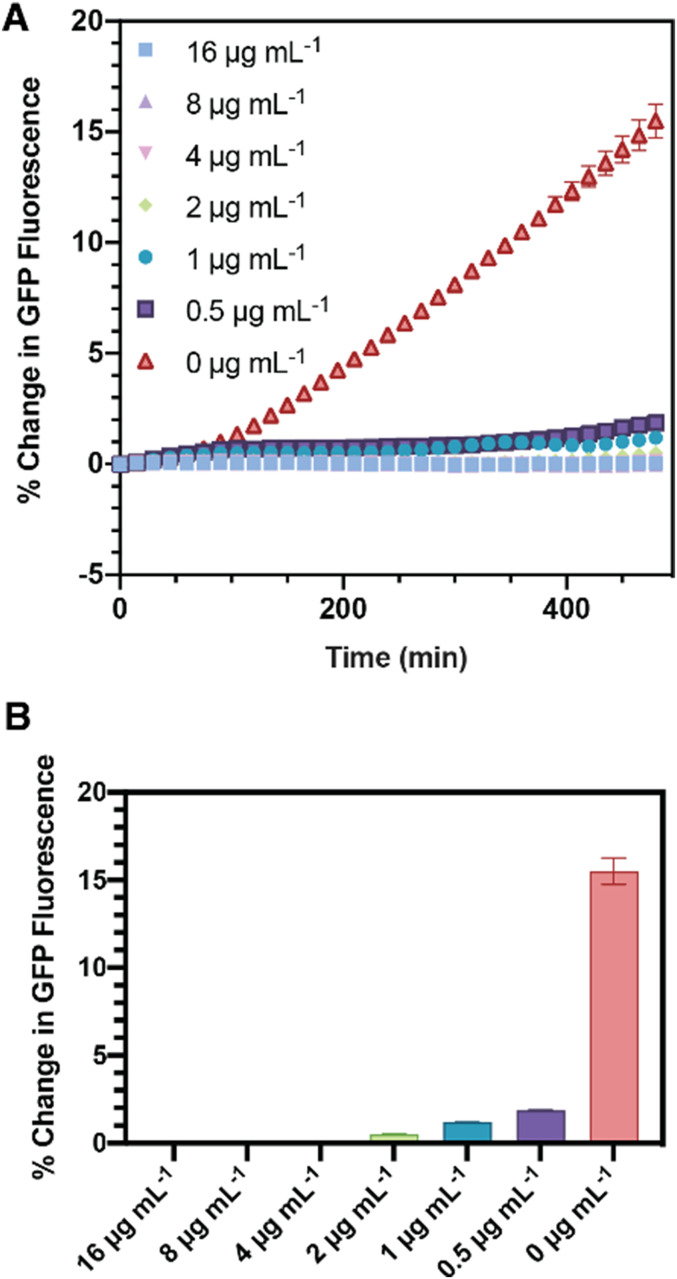
Minimum inhibitory concentration determination in hydrogel plugs. (a) The mean percentage change in integrated fluorescence intensity from *sfgfp*-expressing *E. coli* within the isolation valve with respect to *t* = 0 was measured every 15 min for 8 h for a range of concentrations of ampicillin. (b) Mean change in integrated fluorescence intensity after 8 h. Error bars: ±1 standard deviation, *n* = 5.

Antibiotic susceptibility is typically determined by measuring MIC of an antibiotic on a bacteria culture and is used to identify resistance to different antibiotics and doses are often given in reference to the MIC.^26^ Though there are various methods and tools for measuring MIC,^27^ it is typically performed by cultivating bacteria overnight with a serial dilution of antibiotic in a microtiter well plate. This is a test that is very well-suited to our platform as the valves enable different hydrogel plugs to be incubated with different concentrations of antibiotics on the same chip. Furthermore, we sought to measure the on-chip MIC to identify if small volumes and volume excluded by the hydrogel affected the MIC. *E. coli* were cultivated on-chip in agarose plugs. Ampicillin in varying concentrations (0–16 *μ*g/ml) was delivered to the plugs, which were then isolated by the valves for 8 h. sfGFP fluorescence was monitored over that same period and used to determine bacterial growth, as shown in Fig. 4. As ampicillin is bacteriolytic,^28^ sfGFP fluorescence only provides information on colony growth; we expect sfGFP from lysed cells to remain in the isolation valves until cleared. We found, after 8 h, the MIC of ampicillin to be 4 *μ*g/ml. This is consistent with our own microtiter plate-based MIC measurements (Fig. S1 in the supplementary material) as well as literature. *E. coli* MG1655 has a reported MIC for ampicillin of 8 *μ*g/mL using a conventional microtiter plate method,^29^ and microfluidic methods have reported an MIC of 4–8 *μ*g/ml.^30^

Our main goal was to develop a means of observing the effects of transient dosing of antibiotics on bacteria. The antibacterial effects of antibiotics can be categorized by pharmacokinetic and pharmacodynamic parameters (i.e., serum concentration and minimum bactericidal concentration, respectively) into two broad categories: time dependent and concentration dependent.^31^ In the former, the duration that the dose remains above the MIC is the critical parameter; in the latter, it is the area under curve (AUC) where the antibiotic remains above the MIC. Ampicillin belongs to the time-dependent class of antibiotics where time-above-MIC is the critical parameter. Here, we begin to explore whether this time-dependency holds true at very short durations (10–20 mins) of high concentration doses (approx. 6–25 × MIC) for which the hydrogel-isolation valve platform is well suited to address.

**FIG. 5.**
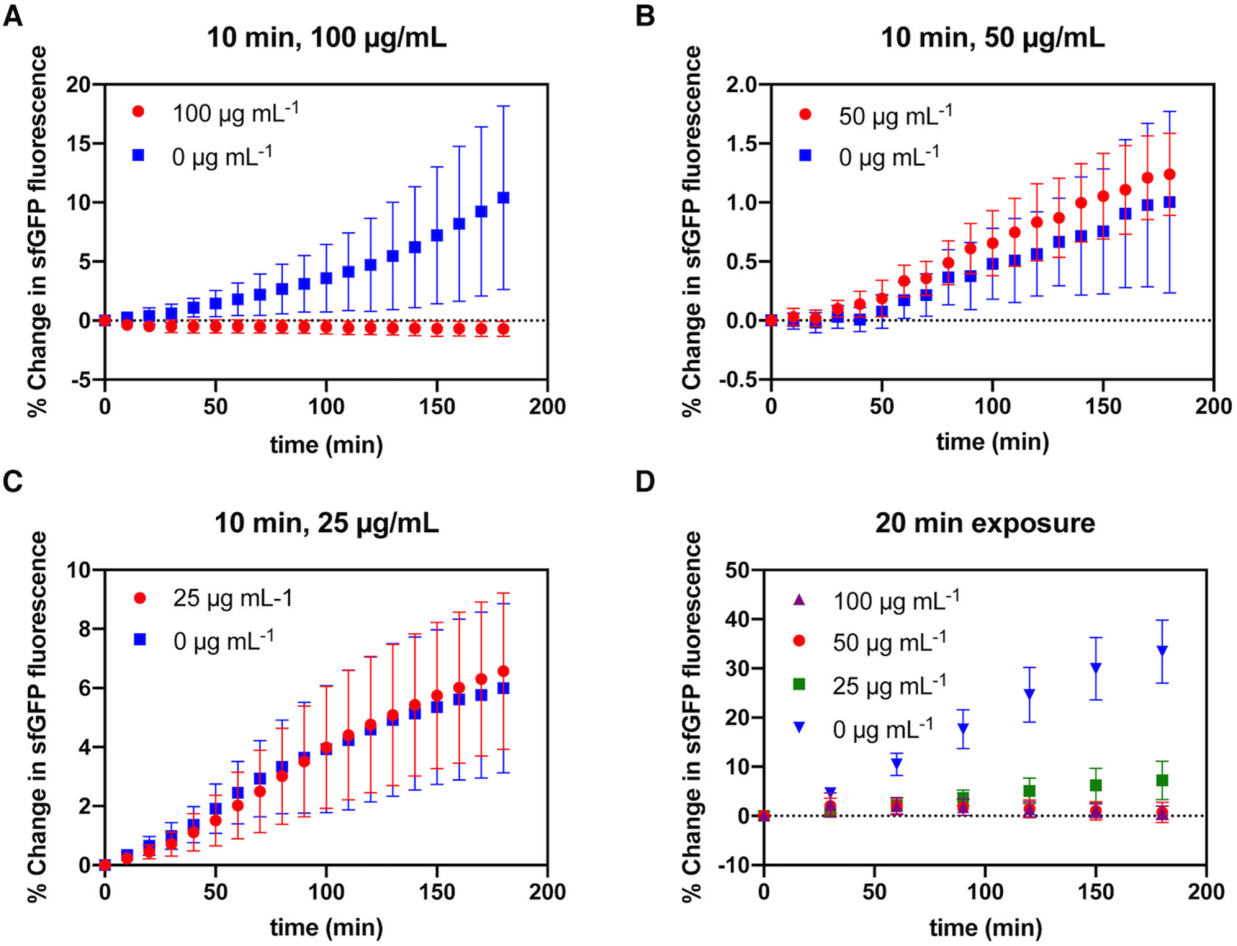
Transient dosing of ampicillin. Mean percentage change in sfGFP mean fluorescence intensity with respect to *t* = 0 from *sfgfp*-expressing *E. coli* in hydrogel plugs exposed to different concentrations of ampicillin for 10 min (a)–(c) or 20 min (d). Fluorescence was recorded after the dosing was complete. Data from individual wells are plotted. Line indicates trend in mean. (a) 10 min exposure to 25 *μ*g ml^−1^ ampicillin; *n* = 8. (b) 10 min exposure to 50 *μ*g ml^−1^ ampicillin; *n* = 8. (c) 10 min exposure to 100 *μ*g ml^−1^ ampicillin; *n* = 7, 0 *μ*g ml^−1^ (control); *n* = 9, 100 *μ*g ml^−1^. (d) 20 min exposure to 25, 50, and 100 *μ*g ml^−1^ ampicillin; *n* = 8.

We compared exposing *E. coli* in agarose hydrogel plugs to 100, 50, 25, and 0 *μ*g/ml of ampicillin for either 10 or 20 min and then monitoring growth over the subsequent 3 h. The maximum killing rate for time-dependent antibiotics has been found to occur at four to five times the MIC,^32,33^ which is comparable to the lowest concentration of ampicillin we chose. Thus, it would be expected that there should be no difference in the bactericidal effect between 10 and 20 min doses. However, our results, shown in Fig. 5, suggest that at such short durations, the bactericidal response depends on both concentration and duration (i.e., AUC). For a 10 min dose, only 100 *μ*g/ml of ampicillin seemed to show a bactericidal effect (p < 0.05). However, a 20 min dose of 100 and 50 *μ*g/ml appeared to have the bactericidal effect as one-way ANOVA and Tukey's post-hoc test did not reveal a significant difference (p = 0.9995). From an AUC perspective, 100 *μ*g/ml ampicillin for 10 min and 50 *μ*g/ml ampicillin for 20 min are equivalent, as both dose profiles resulted in inhibition of bacterial growth. There was also an increase in the bactericidal activity for a concentration of 25 *μ*g/ml ampicillin when exposed for 20 min but not to the same degree as the two higher concentrations. Indeed, it seems to be a critical exposure concentration and duration where cells eventually still grow.

The combination of hydrogels for cell culture and isolation valves to control reagent exposure has allowed us to dose antibiotics in short pulses. This is a meaningful advantage over other microfluidic techniques for studying antibiotic susceptibility and persistence. To our knowledge, the only previous report to study transient dosing of antibiotics exposed bacteria to the antibiotic for periods on the order of hours;^20^ here, we present dosing on the order of tens of minutes. For instance, while droplet-based techniques can reveal heterogeneity among populations of bacteria and do so with high throughput,^34^ adding and then removing antibiotics are difficult. Encapsulating bacteria within hydrogel beads or spheres^35^ provides a possible solution, as gel beads can readily be exchanged from one solution to another. Once the immiscible phase used to generate the gel beads has been removed and the beads have been washed, such gel beads could conceivably be loaded into a microfluidic device for reagent exchange. However, our proposed solution offers a single microfluidic device in which immobilization and antibiotic dosing are executed in a straightforward manner. With the device and method presented here, it is possible to provide short doses of antibiotics, track the cells over time, and compare multiple conditions on the same microfluidic device. While we relied on *E. coli* constitutively expressing sfGFP for quantitative measurements, it is possible to adapt this platform to non-fluorescent strains of bacteria. We demonstrated compatibility with fluorescent dyes for viability (i.e., alamarBlue), but it might also be possible to visually estimate bacteria growth with brightfield microscopy.^36^ We also explored whether it was possible to recapitulate our microscale method on the macroscale by immobilizing *E. coli* in a thin layer of agarose on the bottom of a 96-well plate. While a 20 min exposure could be executed and could achieve similar results (Fig. S2 in the supplementary material), it was difficult to perform all pipetting steps within a shorter time frame (e.g., 10 min). Furthermore, controlling the thickness of the agarose layer in the bottom of a microtiter plate proved difficult, whereas the microfluidic isolation valves provided a consistent geometry of hydrogel.

## CONCLUSION

Small motile cells such as bacteria can be difficult to retain and study over long periods of time. Our microfluidic platform combines hydrogels for bacteria entrapment with isolation valves to control the exposure of bacteria to different concentrations of antibiotics in a pulsed manner. In doing so, the bacteria could be exposed to pulses of antibiotics as short as 10 min, cleared of antibiotic, and subsequently monitored by microscopy. The platform could be employed in future for studies of more complex drug concentration patterns including repeated supply as well as studies on several antibiotics at the same time.

The combination of hydrogels with isolation valves also opens up opportunities for other types of experiments or analyses. The ability to link phenotype to genotype has been a powerful tool for understanding antibiotic resistance and persistence.^37,38^ Though we did not explore this in this report, it could be possible to recover the bacteria for genetic analysis by melting the agarose hydrogel. Beyond microbiology, this platform could also be used with other non-adherent cell types. Alternatively, the hydrogel plug provides an opportunity to cultivate cells in three dimensions while scaling up throughput and automating the experimental control. Isolation-valve-based microfluidic devices have been reported with over 1000 isolation valves;^39^ combined with hydrogel plugs, these devices could be useful for higher throughput screening.

## METHODS

Unless otherwise stated, all reagents were purchased from Sigma Aldrich (Saint Louis, MO, USA).

### Microfluidic device fabrication

A microfluidic device with ring-shaped isolation valves was used to form hydrogel plugs, cultivate bacteria, and deliver antibiotics with a time-dependent dosing profile. The device comprised two layers, a bottom fluidic layer and a top pneumatic layer. In the bottom layer, a single inlet was divided into eight parallel channels, which merged together into a single outlet. The pneumatic layer contained eight sets of eight ring-shaped isolation valves to create a total of 64 isolation chambers. The inner diameter of the isolation valves was 175 *μ*m, confining a volume of approximately 400 pl. The device was made from poly(dimethylsiloxane) (PDMS) (Dow Chemical Company, Midland, MI, USA) by mixing the elastomer with a curing agent in a 10:1 ratio (Sylgard 184, Dow Corning, Midland, MI, USA). Both bottom and top layers were formed over SU-8 (MicroChem, Westborough, MA, USA) moulds on silicon wafers with a feature height of 17 *μ*m. For the bottom layer, approximately 2–3 g of the PDMS mixture was spin-coated on an SU-8 mould resulting in an ∼30 *μ*m thick layer. The top layer was formed by pouring ∼40 g of PDMS onto an SU-8 mould. Both wafers were baked at 80 °C for 2 h. Using a razor blade, the top layer was cut into individual devices and eight 1 mm holes were punched for the pneumatic channels. The top and bottom layers were then aligned and bonded together with an intermediate layer of the curing agent; the bonded PDMS layers were then baked at 80 °C for 2 h. Individual devices were cut out and 1.5 mm holes were punched for inlets and outlets to the fluidic layer. The PDMS devices were bonded to No. 2 thickness (170–250 *μ*m) cover glass (Menzel, Fisher Scientific, Hampton, NH, USA) by plasma bonding.

### Microfluidic device operation

Prior to use, the fluidic channels were filled with 4% (w/v) bovine serum albumin (BSA) in phosphate-buffered saline (PBS; *p*H 7.4) and the pneumatic channels were filled with de-ionized H_2_O by centrifugation. The microfluidic devices were mounted onto the stage of an automated inverted microscope (Nikon Ti2, Nikon, Tokyo, Japan) enclosed in an environmental chamber. Temperature was set to 37 °C at the start of all experiments. A rack of 8 three-way stopcock luerlock valves (Cole-Parmer, Vernon Hills, IL, USA) connected in series was used to connect a single pressurized air supply (2 bar) to the eight pneumatic channels. This allowed each channel to be operated independently. Polytetrafluorethyl (PTFE) tubing (inner diameter 0.25 mm, outer diameter 1.59 mm) was connected directly to the inlet of the microfluidic chip and to a 1 ml plastic syringe. A programmable syringe pump (neMESYS from Cetoni GmbH, Korbussen, Germany, or Aladdin AL-1000 from World Precision Instruments, Friedberg, Germany) was used to control the syringe.

### Bacterial culture

*E. coli* strain K12 MG1655 transformed with pSEVA271-*sfgfp*^24^ was used for the characterization of the microfluidic device. The strain overexpresses super folding green fluorescent protein (sfGFP), enabling quantitation of bacteria. Bacteria were streaked onto lysogeny broth (LB) agar plates (BD Difco LB agar, Miller, Thermo Fisher Scientific, Waltham, MA, USA), and single colonies were cultivated in 2 ml LB culture media supplemented with 50 *μ*g ml^−1^ kanamycin sulfate. Liquid cultures were grown at 37 °C and with a shaking speed of 200 rpm in a shaking incubator overnight. The following day, bacteria were diluted 1:10 in fresh media and grown until optical density at 600 nm (OD_600_) > 0.5.

### Hydrogel plug formation

Agarose hydrogel plugs were formed within the microfluidic device as summarized in Fig. 1(b) and described below. A 3% (w/v) agarose solution was prepared by dissolving ultra-low gelling temperature agarose in sterile-filtered PBS and heating at 80 °C with 1000 rpm shaking in a ThermoMixer (Eppendorf, Hamburg, Germany) until dissolved. The hydrogel was then cooled to 40 °C and diluted to 2.85% (w/v) by the addition of a suspension of *E. coli*, buffer, or solution of fluorophore. Agarose was loaded into the microfluidic device from a reservoir (200 *μ*l pipet tip) by withdrawing the syringe at a rate of 2 *μ*l min^−1^. The valves were closed and wash buffer (100 mM NaCl, 10 mM Tris base, 1 mM EDTA, and 0.1% (v/v) Tween-20) was injected into the chip at 2 *μ*l min^−1^ for 20 min. Temperature control was turned off and the microfluidic device was placed on an aluminum slide (25 × 75 × 2 mm^3^) on ice with a continuous flow (2 *μ*l min^−1^) of either wash buffer or, for experiments with bacteria, LB containing 0.1% (v/v) Tween-20. After 30 min, the microfluidic device was removed and the temperature of the environmental enclosure set to 37 °C.

### Diffusion experiments

A 2.85% (w/v) agarose gel was prepared as above. For experiments monitoring the diffusion of SRB out of the hydrogel plug, the agarose solution also contained 10 *μ*M SRB. Hydrogel plugs were then prepared in the isolation valves on the microfluidic device, as described above. For monitoring diffusion into the hydrogel plug, a 10 *μ*M solution of SRB in PBS was injected for 5 min at a flow rate of 2 *μ*L min^−1^; for monitoring diffusion out of the hydrogel plug, the channel contained PBS without any SRB. After resting for 1 min, the valves were opened. Fluorescence images were taken every 1.5 s for 37.5 s.

### On-chip cell cultivation and imaging

All on-chip cell cultivation experiments began by opening the valves for 1 min and exposing the *E. coli* to a fresh LB medium. Bacteria were then cultivated with closed valves and the LB medium containing 0.1% (v/v) Tween-20 was perfused through the chip at a rate of 1 *μ*l min^−1^. Brightfield and fluorescence images were captured using a Nikon Ti2 automated inverted microscope equipped with a Nikon DS-Qi2 CMOS camera and a SOLA II LED light source for fluorescence excitation. GFP fluorescence was measured with a 470/40 nm excitation filter, a 500 nm dichroic mirror, a 535/50 nm barrier filter, and an exposure time of 50 ms at 10% LED intensity. alamarBlue fluorescence was measured with a 560/40 nm excitation filter, a 595 nm dichroic mirror, a 630/60 nm barrier filter, and an exposure time of 100 ms at 10% LED intensity. SRB fluorescence was measured with a 540/25 nm excitation filter, a 565 nm dichroic mirror, a 605/55 barrier filter, and an exposure time of 50 ms at 10% LED intensity. A 10×  objective was used.

### MIC determination

A simple off-chip MIC assay was performed in a 96-well plate. A stock solution of ampicillin (512 *μ*g ml^−1^) was serially diluted 1:2 in LB containing 50 *μ*g ml^−1^ kanamycin and 0.1% Tween-20. An overnight pre-culture of *E. coli* was diluted 1:100 to OD_600_:0.1 and added in equal volume to the ampicillin-containing wells. Positive (only *E. coli*) and negative (only media) controls were also prepared. The 96-well plate was incubated overnight at 37 °C without shaking. alamarBlue (Thermo Fisher) was prepared as a 1:10 dilution in LB containing 50 *μ*g ml^−1^ kanamycin and 0.1% Tween-20 from the stock and 30 *μ*l was added to each well. The 96-well plate was incubated for a further 2 h at 37 °C without shaking before measuring GFP and alamarBlue fluorescence on a Cytation 5 plate reader (BioTek Instruments Inc., Sursee, Switzerland).

An on-chip MIC assay was performed by injecting LB with 0.1% Tween-20 and 0, 2, 4, 8, and 16 *μ*g ml^−1^ ampicillin using a syringe pump operating at 2 *μ*l min^−1^ into the microfluidic device prepared with *E.coli*-laden hydrogel plugs, as described above. The isolation valves were opened to expose the selected hydrogel plugs to the desired concentration of ampicillin. Valves were opened for 1 min in the presence of the desired concentration of ampicillin and then closed for the remainder of the experiment. The LB medium with 0.1% (v/v) Tween-20 was perfused through the chip at a rate of 1 *μ*l min^−1^. Each concentration of antibiotic was injected for 5 min before opening the selected isolation valves. The MIC was defined as the concentration that resulted in 99% inhibition of bacteria growth.

### Pulsed dosing experiments

*E.coli*-laden hydrogel plugs were prepared as described above. Prior to pulsed dosing experiments, *E. coli* were incubated on-chip at 37 °C for 4 h after exposing to fresh LB media. Solutions of 25, 50, or 100 *μ*g ml^−1^ ampicillin were prepared in LB supplemented with 50 *μ*g ml^−1^ kanamycin and 0.1% Tween-20. For 10 min dosing times, a single chip was used for each concentration of ampicillin and included a control of no ampicillin administered to some of the hydrogel plugs. Ampicillin was administered by injecting the solution at 2 *μ*l min^−1^ for 2 min, waiting for 1 min, opening the valve for 1 min, and closing the valve for 10 min. During this time, LB supplemented with 50 *μ*g ml^−1^ kanamycin and 0.1% Tween-20 was then injected into the device at 2 *μ*l min^−1^ for 5 min. Isolation valves for hydrogel plugs that were to be exposed to 0 *μ*g ml^−1^ ampicillin were opened at this time for 1 min and closed for 10 min. After respective 10 min incubations, valves were opened for 1 min to expose the hydrogel plugs to fresh LB media and then closed again. The LB medium containing 0.1% (v/v) Tween-20 was perfused through the chip at a rate of 1 *μ*l min^−1^. For 20 min incubation with ampicillin, the method was repeated much as described above, except all four concentrations (0, 25, 50, and 100 *μ*g ml^−1^) of ampicillin were dosed on a single chip. In this case, imaging was done with a 4× objective and stitched with a 5% overlap. Ampicillin was added to the microfluidic chip in increasing concentration such that each concentration was added approximately 5 min after the previous. Likewise, the valves were subsequently washed with fresh LB media in 5 min intervals such that the whole procedure took approximately 35 min to deliver and clear all four concentrations of ampicillin. After the hydrogel plugs exposed to 100 *μ*g ml^−1^ ampicillin had been washed with LB, valves were then closed and then brightfield and fluorescence images were taken every 30 min for 3 h. The LB medium containing 0.1% (v/v) Tween-20 was perfused through the chip at a rate of 1 *μ*l min^−1^.

### Data analysis

Fluorescence intensities were measured using Fiji image analysis software.^40^ SRB, FITC-Dextran, and alamarBlue fluorescence intensities were measured using mean fluorescence over the inner circle of the isolation valve. Bacteria growth via GFP was calculated by comparing the percentage change in integrated fluorescence intensity to *t* = 0 for each individual hydrogel plug measured. Microsoft Excel and GraphPad Prism 8 were used to process the data and generate plots. For comparing 10 min doses, multiple t-tests with the Holm-Šídák method was used to compare the antibiotic with the control, performed on the same chip. For 20 min doses, a one-way ANOVA with Tukey's multiple comparisons was used.

## SUPPLEMENTARY MATERIAL

See the supplementary material for results from *off-chip* experiments and the description of the method for off-chip transient dosing.

## Data Availability

The data that support the findings of this study are available from the corresponding author upon reasonable request.
